# Age-associated polyamines in peripheral blood cells and plasma in 20 to 70 years of age subjects

**DOI:** 10.1007/s00726-023-03269-2

**Published:** 2023-06-13

**Authors:** Manuel Sánchez, Lorena Suárez, Gabriela Banda, Eva Barreiro-Alonso, Ignacio Rodríguez-Uña, José Manuel Rubín, Begoña Cantabrana

**Affiliations:** 1 Farmacología, Departamento de Medicina, Facultad de Medicina, Universidad de Oviedo, c/ Julián Clavería 6, 33006 Oviedo, Spain; 2 Instituto Universitario de Oncología del Principado de Asturias (IUOPA), c/ Fernando Bongera s/n, Edificio Santiago, Gascón Campus El Cristo B, 33006 Oviedo, Spain; 3 Instituto de Investigación Sanitaria del Principado de Asturias (ISPA), Av. de Roma s/n, 33011 Oviedo, Spain; 4 Servicio de Digestivo, Hospital Universitario Central de Asturias (HUCA), Av. Roma s/n, 33011 Oviedo, Spain; 5 Fundación de Investigación Oftalmológica (FIO), Instituto Universitario Fernández-Vega (IUFV), Universidad de Oviedo, Av. Dres. Fernández-Vega 34, 33012 Oviedo, Spain; 6 Servicio de Cardiología, Hospital Universitario Central de Asturias (HUCA), Av. de Roma s/n, 33011 Oviedo, Spain

**Keywords:** Polyamines, Human peripheral blood, Age, Putrescine, Spermidine, Spermine

## Abstract

Dietary polyamines have been associated with slowing ageing processes and various pathologies, raising the importance of establishing reference values at different ages throughout life. This study aimed to analyse age-dependent variations in polyamine content using peripheral blood cells and plasma in a healthy and homogeneous population. Peripheral blood of 193 volunteers of both sexes (20–70 years), selected by convenience, was processed to separate cells and plasma. A pre-column derivatization method was used to determine the amines by HPLC (nmol or pmol/mg protein or nmol/ml) to analyse their association with the age (continuous or ordinal in decades) of the subjects. Putrescine and spermine weakly declined significantly in mononuclear cells with age. In erythrocytes and plasma, putrescine showed an evident decrease in the 60–70-year-old group compared to the rest. The ratios between polyamines, mainly in erythrocytes, decreased in the 60–70 years age group and increased the ratio of putrescine in mononuclear cells/erythrocytes. The ratio of putrescine in mononuclear cells/erythrocytes was higher in the 60–70-year-old age group than in the rest. In a sample of subjects (20–29 vs. 60–70 years), whole blood polyamines were not significantly different when differences existed in erythrocytes. Polyamine homeostasis in blood cells and plasma changed with age. Putrescine declined in mononuclear cells and decreased in erythrocytes and plasma in the decade of the 60 s. Further studies should establish an age-dependent phenotype and whether polyamines’ supplementation could restore the decreased values and be associated with long-term overall biological benefits.

**Supplementary Information:**

The online version contains supplementary material available at 10.1007/s00726-023-03269-2.

## Introduction

Ageing constitutes a complex biological process through a combination of genetic bases and environmental factors, which lead to phenotypic and functional changes, resulting in the death of all living organisms. Globally, life expectancy in humans has increased in recent decades, a trend that continues and should be associated with healthy ageing (Robine 2021).

Attention has been given to modifiable factors, including lifestyle changes, to slow down the ageing process. Healthy dietary patterns, generally based on nutritious food groups, have been associated with an improvement of age-related decline in functions and diseases (Petersson and Philippou 2016). Regarding polyamines, epidemiological findings and interventional studies associate higher intakes of polyamine-rich food (Soda et al. 2021) (or specifically one of them: spermidine) (Madeo et al. 2020) with lower morbidity and mortality (Kiechl et al. 2018).

Polyamines are ubiquitous polycationic compounds present in all living cells, subject to highly regulated mechanisms of homeostasis essential to cell growth and proliferation. In animals, they are synthesized de novo from ornithine and converted to putrescine (via the rate-limiting enzyme ornithine decarboxylase), then producing spermidine to form spermine (Wallace 2000). Exogenous sources contributing to maintaining blood levels include dietary intake from various foods (Munoz-Esparza et al. 2019), produced by the intestinal microbiota (Nakamura et al. 2019), bile and pancreatic exocrine secretion, and cellular exfoliation (Loret et al. 2000). These may be affected by different age-related factors (Bloom et al. 2017; Acar Tek and Karacil-Ermumcu 2018).

Their proposed beneficial effects have focused attention on the role of oral supplementation of polyamine-rich foods (Soda et al. 2021), spermidine (Pekar et al. 2020), and the polyamine-producing intestinal microbiota (Nakamura et al. 2019) on human health. Also, the effects focused on the counteraction of some age-associated biological and functional disorders (Pekar et al. 2020; Schroeder et al. 2021), and survival (Kiechl et al. 2018). Although, it must be considered that an increased metabolism of polyamines has also been associated with various pathological conditions (Li et al. 2020; Sagar et al. 2021).

The critical role of polyamines in the organism’s biological functions gives rise to establishing reference values at different ages throughout life. Given the ease of obtaining blood samples in humans, whole blood is mainly used to determine polyamines. However, the determination of polyamines separately in cells and plasma might provide more precise information on the evolution of their values with age. In cells, polyamine homeostasis is tightly regulated (Shantz and Levin 2007). Additionally, physiological ageing is associated with a decline in immune system function (Zhang et al. 2021), being modulated by polyamines (Carriche et al. 2021), and erythrocytes were proposed as markers of various diseases (Pretorius et al. 2016). No studies were conducted to determine polyamines in blood cells and plasma covering a wide range of ages of adulthood in the same population following a Mediterranean dietary pattern, which is considered rich in polyamines (Soda et al. 2021).

Thus, the present study aimed to determine by HPLC the content of polyamines in mononuclear cells, erythrocytes, and plasma of peripheral blood of healthy homogeneous adult populations of different ages, from 20 to 70 years. No older ages were included in the study due to the limited number of subjects lacking diseases or pharmacological treatments that could alter the polyamine content in blood samples unrelated to age. In addition, the polyamine determination was repeated in some participants, and in others, from 20 to 29 and 60 to 70 years, the content of polyamines was also analysed in whole blood.

The data obtained could be used as a non-invasive reference of the normal range of polyamines and to guide the subjects’ levels if a diet rich in polyamines or polyamine supplements were suggested.

## Methods

### Participants and study design

An observational study was conducted on 193 healthy Caucasian volunteers (96 men and 97 women) between 20 and 70 years of age. They were recruited consecutively from March 2012 to July 2016 and from May 2016 to December 2021 (independently of sex) by feasibility among blood donors (Centro Comunitario de Sangre y Tejidos de Asturias, Spain) and via medical consultations, as long as they fulfilled the criteria of an optimal nutritional state, absence of known diseases and pharmacological treatment. The study was explained to participants, who signed an informed consent granting permission to extract (from 9:30 to 12 am, mostly from 9:30 to 11:30) a 4.5 ml blood sample in tubes with EDTA to prevent clotting. They were non-fasting, and the interval between breakfast and the extraction time was, on average, 3 h.

The research was approved by the Comité de Ética de la Investigación del Principado de Asturias (Spain) (reference 28/2010) and was conducted according to the guidelines of the Declaration of Helsinki.

The variables considered were age (continuous or ordinal, as groups of age by decades), sex and the biogenic amines in peripheral blood samples, determined by high-performance liquid chromatography (HPLC).

### Separation of cells and plasma from peripheral blood

The blood samples were taken to the laboratory in less than an hour to separate the blood cells and plasma with Ficoll, following the manufacturer’s protocol (GE Healthcare Ficoll-Paque PLUS). Cells were determined by using a Neubauer counting chamber. In 8 participants from 20 to 29 years of age and 12 from 60 to 70 years, 200 µl whole blood aliquots were also obtained. Afterwards, the aliquots of mononuclear cells, erythrocytes, plasma, and whole blood were frozen in liquid nitrogen to be maintained at – 80 °C until used to determine the content of the amines.

### Determination of polyamines via HPLC

As previously described (Suárez et al. 2019), the amines were determined using a pre-column derivatization method in the obtained samples. To the whole pellet of mononuclear cells and the 50 µl of the aliquot of erythrocytes was added 300 µl and 250 µl of water (Milli-Q), respectively, of the samples of plasma, were taken 450 µl. These samples were homogenized and treated with perchloric acid to reach a final concentration of 15.8% for 10 min at 4 °C. Then, the samples were processed and chromatographed in an HPLC (Shimadzu Prominence) using a C18 (2.5 μm, 3.0 × 75 mm) reverse-phase column (XBridge from Waters), at room temperature 22–24 °C. The determinations of amines in mononuclear cells were expressed as nmol/mg of protein and the erythrocytes and plasma as pmol/mg of protein; the units to calculate the ratios of polyamines between cells and plasma were nmol/mg of protein in all cases. The protein content was determined according to the Bradford procedure by taking 50 µl of the homogenized sample. The polyamines content in blood cells was also normalized per million cells. In whole blood, the polyamines content was expressed as nmol/ml.

### Chemicals

The compounds used putrescine (tetramethylenediamine), spermidine (N-(3-aminopropyl)-1,4-butanediamine), spermine (N,N′-bis(3-aminopropyl)-1,4-butanediamine), N-acetylputrescine hydrochloride and 2-hydroxydiaminopropane (purchased from Sigma–Aldrich), were dissolved in purified water (10–15 MΩ·cm).

### Statistical analysis

The sample size (*n*) was determined by the number of participants needed to perform a multiple regression analysis. ﻿The simple way to calculate this is n ≥ 50 + (8 × M), where M is the number of independent or explanatory variables (Tabachnick and Fidell 2013). These were three polyamines and N-acetylputrescine in three blood samples (mononuclear cells, erythrocytes and plasma), resulting in* n* > 146 participants, from 20 to 70 years of age.

Non-parametric statistics were used since normality was violated. Thus, Spearman’s rank correlation (*r*_*s*_) (two-tailed) was used to determine the association between variables (referred to as weak, up to 0.3, moderate, from ~ 0.4 to 0.6, and strong from ~ 0.7 to 0.9). Box-and-whisker plots were used to show the concentrations of biogenic amines in the five ordinal groups of ages by decades, whose statistical significance was calculated using the Kruskal–Wallis test (pairwise comparison *p*-values adjusted by the Bonferroni correction for multiple tests). The Mann–Whitney *U*-test analysed the differences between two independent groups of age (*p*-values adjusted by Bonferroni correction).

Multiple regression analysis was performed to explore the relationship between the age in years, the dependent variable, and the main polyamines determined in blood cells and plasma as potential predictor variables.

For all analyses, values of *p* ≤ 0.05 were considered significant. These were performed using IBM SPSS^©^ Statistics version 27.0 (IBM Corp.).

## Results

### Participants in the study

The median age was 45 years, 47 for men (*n* = 88, 51.2%) and 38 for women (*n* = 84, 48.8%), respectively. They were categorically distributed by age groups in decades (Table 1).

**Table 1:** Participants and polyamines content in samples of peripheral blood

Groups (years)	20–29	30–39	40–49	50–59	60–70	Kruskal–Wallis-test	30–59
*n* (Gender: M/F)	41 (10/31)	39 (15/24)	40 (28/12)	36 (22/14)	37 (21/16)		115 (65/50)
Age (Years)	24 (21–26.5)	36 (34–38)	45 (43–47)	54.5 (51–57)	63 (61–66.72)		45 (38–51)
Mononuclear Cells (nmol/mg of protein)							
Putrescine	1.03 (0.77–1.36)	1.03 (0.77–1.64)*	0.99 (0.7–1.25)	0.87 (0.61–1.12)	0.76 (0.67–1.01)	*H*(4) = 12.01, *p* = 0.017	0.97 (0.7–1.24)^†^
Spermidine	5.02 (3.95–6.22)	4.3 (3.22–5.9)	4.49 (3.57–5.75)	3.86 (3.1–4.82)	3.82 (2.91–5.11)	*H*(4) = 12.16, *p* = 0.016	4.18 (3.32–5.53)
Spermine	8.52 (6.11–11.85)	8.96 (6.7–12.16)*	8.64 (6.97–12.92)*	7.5 (5.45–8.97)	7.17 (5.48–8.42)	*H*(4) = 15.35, *p* = 0.004	8.15 (6.41–11.86)^††^
N-acetylputrescine	2.72 (1.51–4.47)	2.1 (1.45–3.16)	1.96 (1.59–2.5365)	1.85 (1.67–2.45)	1.64 (1.12–2.79)	*H*(4) = 6.13, *p* = 0.190	2 (1.4–2.69)
Erythrocytes (pmol/mg of protein)							
Putrescine	10.19 (5.64–19.43)**	17.51 (10.55–32.8)***^$^	19.28 (11.8–28.63)***^$^	22.92 (13.4–31.35)***^$$^	5.26 (3.89–5.97)	*H*(4) = 65.47, *p* < 0.001	21.13 (11.50–30.20)^†††^
Spermidine	141.86 (93.7–177)	110.61 (85.29–134.3)	121.95 (97.65–131.11)	127.9 (101.3–169.6)	127.18 (106.94–144.89)	*H*(4) = 8.73, *p* = 0.068	117.82 (96.42–147.92)
Spermine	52.57 (36.88–80.79)	60.83 (44.31–90.47)	51.03 (38.25–70.3)	51.12 (37.87–82.82)	45.49 (34.08–77.06)	*H*(4) = 4.31, *p* = 0.366	55.3 (40.95–75.57)
N-acetylputrescine	50.67 (32.87–198.27)	34.25 (28.87–40.63)	33.82 (28.72–44.87)	39.45 (27.01–68.94)	41.85 (34.29–54.95)	*H*(4) = 11.30, *p* = 0.023	35.79 (28.74–45.92)
Plasma (pmol/mg of protein)							
Putrescine	18.65 (14.08–31.17)***	25.74 (16.9–32.29)***	24.25 (14.45–29.22)***	22.79 (13.49–31.35)***	8.36 (7.06–13.75)	*H*(4) = 39.73, *p* < 0.001	24.84 (14.6–31.34)^†††^
Spermidine	10.39 (7.83–13.34)	6.57 (5.51–8.19)^$^	7.2 (5.94–8.59)	7.58 (4.93–9.87)	8.38 (4.88–13.46)	*H*(4) = 11.55, *p* = 0.021	6.92 (5.44–8.58)
Spermine	4.33 (2.08–5.7)*	3.48 (2.9–4.99)	3.87 (2.99–5.95)**	3.47 (2.99–4.9)	2.19 (1.77–4.15)	*H*(4) = 13.28, *p* = 0.009	3.54 (2.99–5.1)^†††^
N-acetylputrescine	538.3 (352–847.6)***	581.4 (476.8–675.4)***	569.5 (517.4–766.5)***	582.4 (516.3–696.6)***	325.5 (269.7–476.4)	*H*(4) = 32.29, *p* < 0.001	579.9 (510.9–721.1)^†††^

Number (*n*) of participants, median of age in years and values of polyamines (nmol or pmol/mg of protein) (Percentile 25th–75th) in peripheral blood cells and plasma in each categorical group by decades and of participants from 30 to 59 years of age

*p ≤ 0.05

**p ≤ 0.01

***p ≤ 0.001 vs. 60–70 years old

^$^*p* ≤ 0.05

^$$^*p* ≤ 0.01

^$$$^*p* ≤ 0.001 vs. 20–29 years old by means of Kruskal–Wallis (KW)-test

^†^*p* ≤ 0.05

^††^*p* ≤ 0.01

^†††^*p* ≤ 0.001 comparing 30–59 vs. 60–70 years old by means of Mann Whitney *U*-test

### Correlation between polyamines and the age of the participants. Median concentrations of polyamines in blood cells and plasma by age groups

**Fig. 1:**
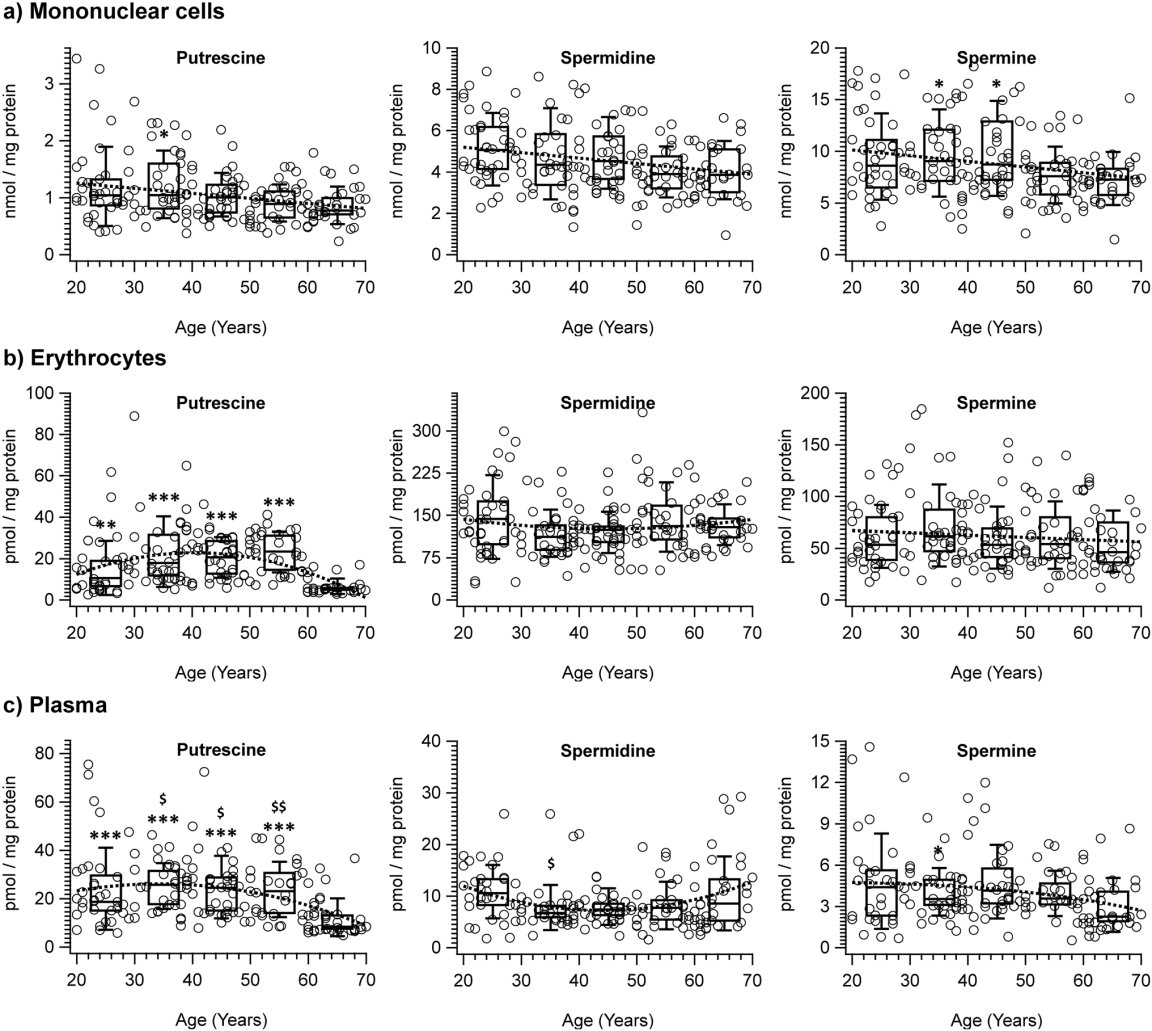
Polyamines content in samples of cells and plasma of peripheral blood. Linear plots and fittings (dotted lines) of the values (nmol/mg of protein) of putrescine (**a**), spermidine (**b**) and spermine (**c**) vs. the participants’ age and box-and-whisker plots regarding the age by decades in the mononuclear cells, erythrocytes, and plasma; *P < 0.05 and **P < 0.01 by comparison with the group of 60–70 years of age; ^$^*p* ≤ 0.05 and ^$$^*p* ≤ 0.01 by comparison with the group of 20–29 years, using Kruskal–Wallis test

Regarding age, in mononuclear cells, putrescine (*r*_*s*_ = − 0.195, *p* = 0.007) and spermine (*r*_*s*_ = − 0.211, *p* = 0.003) (Fig. 1), there was a weak but statistically significant linear decrease in content with age. The Kruskal–Wallis test showed significant differences between independent age groups for putrescine, spermidine, and spermine. The pairwise comparisons with adjusted *p*-values showed significant differences for putrescine with 60–70 years of age being lower than the values of the 30–39 years, and for spermine with 60–70 years of age being lower than the value obtained in the 30–39 and 40–49 years (Fig. 1; Table 1). No differences existed for spermine and N-acetylputrescine (Table 1). The putrescine/spermidine and spermidine/spermine ratios in these cells showed a decrease and an increase in the 60–70 years group compared with the 30–39, respectively (Table 2).

**Table 2:** Ratios between polyamines content in blood cells and plasma of peripheral blood

Groups (years)	20–29	30–39	40–49	50–59	60–70	Kruskal–Wallis-test	30–59
Mononuclear cells							
Putrescine/Spermidine	0.22 (0.17–0.26)	0.24 (0.22–0.28)	0.21 (0.17–0.27)	0.22 (0.19–0.29)	0.2 (0.16–0.26)^**ɸ**^	*H*(4) = 10.98, *p* = 0.027	0.23 (0.19–0.28)^†^
Spermidine/Spermine	0.55 (0.47–0.62)	0.5 (0.45–0.54)	0.5 (0.45–0.55)	0.515 (0.47–0.58)	0.56 (0.5–0.63)^**ɸ**^	*H*(4) = 13.82, *p* = 0.008	0.51 (0.46–0.56)^††^
Putrescine/N-acetylput	0.47 (0.18–0.63)	0.6 (0.43–0.67)	0.48 (0.38–0.65)	0.45 (0.26–0.63)	0.46 (0.23–0. 7)	*H*(4) = 4.63, *p* = 0.326	0.49 (0.38–0.66)
Spermidine/ N-acetylput	1.85 (1.12–2.75)	2.25 (1.62–2.82)	2.34 (1.96–2.86)	2.12 (1.38–2.41)	2.27 (1.36–2.97)	*H*(4) = 4.68, *p* = 0.321	2.18 (1.72–2.81)
Erythrocytes							
Putrescine/Spermidine	0.07 (0.05–0.17)**	0.15 (0.09–0.29)***^$^	0.15 (0.09–0.27)***^$^	0.16 (0.09–0.25)***^$^	0.04 (0.03–0.05)	*H*(4) = 61.88, *p* < 0.001	0.15 (0.09–0.27)^†††^
Spermidine/Spermine	2.44 (1.6–3.28)	1.83 (1.44–2.31)	2.27 (1.76–3)	2.46 (1.65–3.36)^**ɸ**^	2.89 (1.81–3.4)^**ɸɸ**^	*H*(4) = 14.94, *p* = 0.004	2.2 (1.6–2.97)^†^
Putrescine/N-acetylput	0.21 (0.06–0.35)	0.48 (0.28–0.87)***^$$$^	0.48 (0.25–0.76)***^$$^	0.44 (0.23–0.86)***^$^	0.1 (0.07–0.16)	*H*(4) = 50.43, *p* < 0.001	0.46 (0.26–0.81)^†††^
Spermidine/N-acetylput	2.21 (0.97–3.98)	3.2 (2.56–3.71)	3.14 (2.35–4.11)	2.99 (2.07–4.49)	2.64 (1.82–4.21)	*H*(4) = 4.98, *p* = 0.289	3.15 (2.35–4)
Plasma							
Putrescine/Spermidine	1.86 (1.12–3.4)	3. 7 (2.01–5.09)***^$^	2.78 (1.84–4.47)***	2.78 (2.01–5.1)***	1.42 (0.72–1.81)	*H*(4) = 29.94, *p* < 0.001	3.19 (2–5.02)^†††^
Spermidine/Spermine	2.37 (1.7–5.43)	1.84 (1.3–2.25)**	1.74 (1.29–2.16)***^$^	2.1 (1.55–2.56)	2.61 (2.04–5.49)	*H*(4) = 22.72, *p* < 0.001	1.8 (1.34–2.35)^†††^
Putrescine/N-acetylput	0.034 (0.025–0.042)	0.042 (0.029–0.055)**	0.034 (0.027–0.05)	0.039 (0.027–0.051)*	0.026 (0.02–0.033)	*H*(4) = 15.04, *p* = 0.004	0.038 (0.028–0.052)^†††^
Spermidine/N-acetylput	0.019 (0.01–0.035)	0.011 (0.009–0.015)***	0.01 (0.009–0.016)***	0.013 (0.01–0.018)**	0.021 (0.014–0.042)	*H*(4) = 30.81, *p* < 0.001	0.012 (0.009–0.016)^†††^
Mononuclear cells/erythrocytes							
Putrescine	68.64 (46.33–168.88)	52.97 (30.3–133.79)***	49.43 (31.77–98.32)***	39.86 (23.31–63.3)***^$$^	166.54 (105.7–194.6)	*H*(4) = 44.12, *p* < 0.001	45.91 (30.25–91.25)^†††^
Spermidine	37.66 (21.36–53.31)	40.19 (29.01–62.3)	38.36 (28.49–64.59)	30.16 (17.95–38.76)	32.89 (25.57–37.55)	*H*(4) = 13.71, *p* = 0.008	34.75 (23.18–47.99)
Spermine	132.91 (94.07–295.46)	146.98 (79.03–235.76)	158.96 (112.15–335.67)	119.41 (81.5–212.08)	134.69 (88.9–204.71)	*H*(4) = 4.33, *p* = 0.362	143.51 (89.8–233.03)
Mononuclear cells/plasma							
Putrescine	52.93 (32.67–80.11)	41.38 (30.01–69.76)**	44.95 (30.27–73.62)**	45.65 (27.23–59.04)***	86.45 (45.89–114.29)	*H*(4) = 20.38, *p* < 0.001	44.39 (30.05–67.07)^†††^
Spermidine	468.9 (299.69–661.91)	604.39 (459–905.16)	633.29 (524.45–870.73)	560.46 (341.92–827.27)	489.12 (305.84–713.98)	*H*(4) = 11.98, *p* = 0.017	631 (447.56–884.15)^†^
Spermine	2199 (935–3231)	2198 (1613–3770)	2285 (1822–3820)	2246 (1361–2620)	2898 (1620–4566)	*H*(4) = 5.81, *p* = 0.213	2246 (1640–3330)
Erythrocytes/Plasma							
Putrescine	0.51 (0.33–0.93)	0.85 (0.34–1.3014)	0.81 (0.52–1.12)	1.013 (0.66–1.55)*^$$^	0.64 (0.45–0.81)	*H*(4) = 14.79, *P* = 0.005	0.86 (0.52–1.28)^††^
Spermidine	12.65 (9.14–22.07)	17.47 (11.27–23.9)	15.68 (10.61–22.06)	16.41 (12.38–22.43)	15.62 (8.51–27.2)	*H*(4) = 3.72, *p* = 0.445	16.36 (11.77–23.16)
Spermine	12.98 (7.84–20.66)	16.83 (10.88–24.94)	14.69 (7.15–23.74)	15.66 (9.08–21.25)	17.34 (11.04–29.44)	*H*(4) = 5.37, *p* = 0.251	15.3 (9.66–21.41)

Median (Percentile 25th–75th) of the ratios (nmol/mg protein) between polyamines content in mononuclear cells, erythrocytes, and plasma in each categorical group by decades and of participants from 30 to 59 years of age

*N-acetylput* N-acetylputrescine

*p ≤ 0.05

**p ≤ 0.01

***p ≤ 0.001 vs. 60–70 years old

^$^*p* ≤ 0.05

^$$^*p* ≤ 0.01

^$$$^*p* ≤ 0.001 vs. 20–29 years old

^ɸ^*p* ≤ 0.05 vs. 30–39 by means of Kruskal–Wallis (KW)-test

^†^*p* ≤ 0.05

^††^*p* ≤ 0.01

^†††^*p* ≤ 0.001 comparing 30–59 vs. 60–70 years old by means of Mann Whitney *U-*test

In erythrocytes, no linear correlation existed between putrescine, spermidine, and spermine with the age of the subjects. The Kruskal–Wallis test showed significant differences between independent age groups for putrescine and N-acetylputrescine, but not for spermidine or spermine, and the ratios of putrescine/spermidine, putrescine/N-acetylputrescine, and spermidine/spermine. The pairwise comparisons with adjusted *p*-values showed significant differences among the groups in the content of putrescine and putrescine/spermidine and putrescine/N-acetylputrescine ratios, showing an inverted U shape with a significantly lower value in the 60–70 years of age (Fig. 1; Table 1).

The polyamines analysed did not linearly correlate with age in plasma samples (Fig. 1). The Kruskal–Wallis test showed significant differences in the content of amines and their ratios between the independent age groups. The pairwise comparisons with adjusted *p*-values showed significant differences between groups for putrescine and putrescine/spermidine, showing an inverted U shape with significantly lower values in the 60–70 years age group than the rest of the groups. Spermidine and spermidine/spermine ratio also showed a U shape, with significantly lower values for spermine in the 30–39 age group vs. 20–29, and the 30–39 and 40–49 lower than the 60–70 years age group. Spermine showed lower values for the 60–70 years age group than 20–29 and 40–49, N-acetylputrescine was lower in the 60–70 years age group than all other groups. The 60–70 years age group showed lower values for the putrescine/N-acetylputrescine ratio than 30–39 and 50–59, and spermine/N-acetylputrescine ratio than those above 30 years of age (Fig. 1; Table 1).

Similar findings were observed by normalizing the polyamine content in blood cells by referring to the number of cells (Supplementary Table 1). Regarding sex, erythrocytes of males showed significantly lower levels of spermidine (in the 20–29 and 50–59 age groups) and N-acetylputrescine (in the 40–49 age group) than in females, by Mann–Whitney *U*-test (Supplementary Table 2).

### Ratios of polyamines content in mononuclear cells/erythrocytes, mononuclear cells/plasma and erythrocytes/plasma regarding the groups of age

The ratios between putrescine mononuclear cells/erythrocytes and mononuclear cells/plasma showed a U shape with lower values in the three groups comprising 30 to 59 years compared to the 60–70 years age group. However, the ratio of putrescine in erythrocytes/plasma showed higher content in the 50–59 years age group than in the 20–29 and 60–70 years age groups (Table 2).

### Median values of polyamines in subjects from 30 to 59 years of age and comparison with the group of 60–70 years of age

Overall, the analysis showed that the polyamines were more stable from 30 to 59 years of age. Thus, the values obtained were compared with the 60–70 years age group. The Mann–Whitney *U*-test, comparing the polyamines of 60–70 years subjects with those of 30–59, as a group, showed a significant decrease in the content of putrescine and spermine in mononuclear cells, putrescine in erythrocytes and putrescine, spermine and N-acetylputrescine in plasma (Table 1).

There are also significant differences in the ratios between polyamines, decreasing the putrescine/spermidine ratios in mononuclear cells, erythrocytes, and plasma; putrescine/N-acetylputrescine ratios in erythrocytes and plasma; spermidine in mononuclear cells/plasma and the ratio of putrescine in erythrocytes/plasma. In contrast, the ratios of spermidine/spermine in mononuclear cells and erythrocytes and plasma spermidine/spermine and spermidine/N-acetylputrescine increased. Finally, the ratio of putrescine in mononuclear cells/erythrocytes also increased (Table 2).

### Multiple regression analysis of age prediction based on the content of polyamines in mononuclear cells, erythrocytes, and plasma of peripheral blood

The analysis showed that the model contained polyamines of blood cells and plasma as independent variables that significantly contributed as potential predictors of the dependent variable, the age in years. The proportion of variance explained by the model increased with the age of participants from 13.3% when all participants were considered, from 20 to 70 years, to 35.2%, 39.77% and 44.98%, respectively, from 30 to 70 years, 40 to 70 and 50 to 70 years.

Considering the subjects from 40 to 70 years, the equation obtained was: *Y*_(age in years)_ = 66.295 (Constant) + (− 0.847 × − 0.271 putrescine (pmol/mg protein) in erythrocytes) + (− 0.878 × spermine (pmol/mg protein) in plasma) + (0.344 × spermidine (pmol/mg protein) in plasma) + (− 1.111 × spermidine (nmol/mg protein) in mononuclear cells) + (− 0.132 × putrescine (pmol/mg protein) in plasma) (Supplementary Table 3).

### Values of polyamines in whole blood, blood cells, and plasma of peripheral blood in subjects from 20 to 29 and 60 to 70 years of age

Comparing the main polyamines content in whole blood between the group of 20–29 (*n* = 8) vs. 60–70 (*n* = 12) years of age, by means of the Mann–Whitney *U*-test, showed no significant differences, while in the same subjects, spermidine in mononuclear cells (median and 25th–75th percentile) (4.84 (3.97–7.39) vs. 3.68 (2.81–3.88), respectively, *U* = 20, *p* = 0.05) and putrescine (5.58 (4.1–6.7) vs. 4.22 (3.74–4.61), respectively, *U* = 22, *p* = 0.047) and spermidine (160.57 (124.2–180.95) vs. 120.49 (106.56–137.7), respectively, *U* = 21, *p* = 0.038) in erythrocytes were significantly lower in the 60–70 years group than in 20–29 years (Supplementary Table 4).

## Discussion

Overall, the results showed that polyamines in adults, with individual variability, decreased with age in blood cells and plasma. Putrescine was the most relevant decreased polyamine in erythrocytes and plasma between 60 and 70 years. The findings are in line with previous statements of an age-related decline in polyamines, although they differ in the samples tested and the type of polyamine primarily modified. The content of polyamines in cells and peripheral blood plasma can serve as a reference for the values to be maintained or achieved, especially over 60 years of age.

The determination of polyamines in peripheral blood has been used in different studies in humans since these samples are easy to obtain non-invasively. Several studies reported the ageing changes of polyamines or the effect of their intake on blood content, determining them in whole blood. Additional information could be obtained if they were determined separately in plasma and blood cells. They could be more stable in blood cells since polyamine homeostasis is tightly regulated in cells, although their concentrations can be altered by various physiological and pathological factors, including ageing. For this reason, we chose to analyse the polyamines in blood components separately, plasma and cells, to establish their relationship with the age of the subjects. In fact, in a sample of participants, to compare polyamine values between subjects aged 20–29 years versus those aged 60–70 years, the content in erythrocytes and plasma was more sensitive to changes due to age than in whole blood.

The linear correlation analysis between the age and the content of polyamines in the blood components showed that putrescine and spermine gradually decreased in mononuclear cells, with minor variations between age groups. These are similar qualitative findings to the reported age-dependent polyamines decrease in human peripheral blood mononuclear cells (Alsaleh et al. 2020). In erythrocytes and plasma, putrescine showed an inverted U shape, with the lowest value in the 60–70 years age group. The value of spermine in plasma at 60–70 years age group is also lower.

Nevertheless, there were individual variabilities in the content of polyamines, despite minimizing influential factors as much as possible. Thus, the blood sample was always taken in the morning to avoid the influence of the circadian rhythm on the levels of polyamines (Zwighaft et al. 2015). All the participants belonged to the same geographical area. The study was conducted on non-fasting healthy Caucasian people (or at least without known diseases and optimal nutritional state) with a similar sociocultural environment and Mediterranean-type eating habits. However, there could be variabilities in their polyamine intake and the polyamine-producing microbiota (Sittipo et al. 2019), facts not investigated in the present study.

Several factors have been associated with the polyamine decline, including a reduced de novo intracellular synthesis, partly due to the decrease in ODC activity (Nishimura et al. 2006). This reduction could be compensated by the uptake of polyamines from extracellular sources (Nishimura et al. 2006). However, a decrease in food intake, alterations in the microbiota (also influenced by diet) and intestinal absorption that can occur in elderly populations are facts that can compromise the homeostasis of polyamines.

According to our results, the polyamine levels were not uniformly maintained with age. This could be explained by the unequal changes in the synthesis and catabolic pathways of polyamines by aging (Uemura et al. 2020). These might be responsible for the mild changes in the putrescine/spermidine and spermidine/spermine ratios shown in mononuclear cells. And for the modifications in erythrocytes and plasma with a type of inverted U, with lower values in the group from 60 to 70 years of age. Aging affected more polyamine content in erythrocytes than in mononuclear cells, as shown by the substantial variations in the ratio of putrescine content between these cells showing a U shape, with higher values in the group from 60 to 70 years of age. An absence of a uniform trend in the age-related decrease in diverse organs, tissues, and fluids has also been reported (Das and Kanungo 1982) in experimental animals and humans (using post-mortem tissues or whole blood).

Based on the determination of polyamines, the potential predictors of age seem favourable above 30–40 years of age. Putrescine content in erythrocytes has the most significant negative influence on age, being the only one when considering from 50 to 70 years. Interestingly, the polyamine content in the erythrocytes has been proposed as a reference for normal growth (Quemener et al. 1991) and several pathologies’ stages (Pretorius et al. 2016). Besides, spermine and spermidine in plasma, spermidine in mononuclear cells and putrescine in plasma showed a negative relationship with age. Therefore, accordingly, an increase in these polyamines could determine a decrease in age. These findings agree with the age-related decline in polyamines associated with the cellular ageing phenotype, remaining high in the longevity population (Pucciarelli et al. 2012).

Determining polyamines in blood cells and plasma could have advantages over determinations in whole blood, where variations with age were less evident, according to the results obtained using a sample of the subjects.

The sum of the evidence in previous studies supports the supplementation of polyamines, specifically spermidine, to prevent or slow down age-dependent biological changes, protecting the cardiovascular and immune systems and cognition (Soda 2022). However, the amount of intake and blood levels are unknown. In addition, safety concerns may limit intervention studies in humans, as increased polyamines have been associated with pathology, partially through metabolism to cytotoxic agents (Pegg 2013; Igarashi et al. 2018).

Therefore, it seems essential to establish physiological values of polyamines and their ratios in blood cells and plasma to assess the population and have a reference to achieve if dietary polyamine supplementation was considered necessary, which was the aim of our study. Besides, it is essential to study their use as complementary predictive biomarkers to serve as an ageing and frailty index potentially.

Further studies should establish whether long-term supplementation of polyamines or polyamine-rich foods could increase polyamine content in blood cells and plasma and restore the modified ratios between polyamines. Moreover, whether they are associated with long-term overall biological benefits on organisms, as could be expected (Soda 2022; Nakanishi and Cleveland 2021). Also suggested by the finding that spermidine supplementation recovers the autophagy level and function of human T cells from old donors (Alsaleh et al. 2020).

The best way to confirm age-associated changes in polyamine content is through a multi-year longitudinal study, which is not as feasible. In addition, the present study has some limitations since the randomized process was not followed, although guarantees were taken to recruit the subjects consecutively and avoid bias as far as possible.

Furthermore, it was not designed to analyse gender differences or dietary habits to establish the intake of polyamines as responsible for the observed changes in peripheral blood. Despite this, differences could exist in the content of spermidine and N-acetylputrescine in erythrocytes in certain groups of age, being lower in males than in females.

## Supplementary Information

Below is the link to the electronic supplementary material.

Supplementary file1 (DOCX 27 KB)

Supplementary file2 (DOCX 28 KB)

Supplementary file3 (DOCX 43 KB)

Supplementary file4 (DOCX 39 KB)

## Data Availability

The data that support the findings of this study are available from the corresponding author (MS), upon request.
