# Supplementary material for: Age-associated polyamines in peripheral blood cells and plasma in 20 to 70 years of age subjects
Source: Amino Acids. 2023 Jun 13;55(6):789–98. doi: 10.1007/s00726-023-03269-2 (PMC10287822; doi:10.1007/s00726-023-03269-2)
Supplement: Supplementary file 1 — Supplementary file1 (DOCX 27 KB) [file 726_2023_3269_MOESM1_ESM.docx]

**Supplementary Table 1** Participants and polyamines content in samples of peripheral blood. Number (*n*) of participants, median of age in years and values of polyamines (nmol or pmol/mg per million cells) (Percentile 25th-75th) by sex (Male and Female) in peripheral blood cells and plasma in each categorical group by decades.

| **Groups (years)** | **20-29** | **30-39** | **40-49** | **50-59** | **60-70** | **Kruskal-Wallis-test** |
| --- | --- | --- | --- | --- | --- | --- |
| ***n* (Gender: Male/Female)** | 41 (10/31) | 39 (15/24) | 40 (28/12) | 36 (22/14) | 37 (21/16) |  |
| **Age (Years)** | 24 (21-26.5) | 36 (34-38) | 45 (43-47) | 54.5 (51-57) | 63 (61-66.72) |  |
| **Mononuclear Cells (nmol/million cells)** | |  |  |  |  |  |
| Putrescine | 0.425 (0.32-0.6) | 0.48 (0.38-0.59)^***^ | 0.46 (0.36-0.62)^*^ | 0.42 (0.3-0.55) | 0.33 (0.25-0.43) | *H*(4)=18.43, *P*=0.001 |
| Spermidine | 1.88 (1.6-2.43) | 1.79 (1.52-2.47) | 1.9 (1.7078-2.5244) | 1.84 (1.37-2.19) | 1.66 (1.31-2.06)) | *H*(4)=8.61, *P*=0.072 |
| Spermine | 3.41 (2.786-4.58) | 3.78 (3.19-4.62)^***^ | 3.8 (3.13-5.35)^**^ | 3.49 (2.79-3.89) | 2.93 (2.44-3.66) | *H*(4)=19.22, *P*<0.001 |
| N-acetylputrescine  N | 1.07 (0.68-1.9) | 0.9 (0.72-1.28) | 0.86 (0.77-1.25) | 0.9 (0.67-1.32) | 0.67 (0.49-1.35) | *H*(4)=8.08, *P*=0.088 |
| **Erythrocytes (pmol/million cells)** | |  |  |  |  |  |
| Putrescine | 1.67 (0.98-2.93)^**^ | 2.79 (1.95-4.91)^**$^ | 3.26 (1.49-3.96)^***^ | 3.69 (1.66-4.52)^***$$^ | 0.73 (0.66-1.1) | *H*(4)=64.27, *P*<0.001 |
| Spermidine | 19.74 (14.43-29.21) | 17.91 (15.05-22.63) | 17.81 (12.69-22.97) | 20.33 (16.6-28.4) | 20.22 (16.69-24.52) | *H*(4)=5.92, *P*=0.205 |
| Spermine | 7.57 (5.81-10.65) | 10.89 (7.55-15.38) | 7.56 (5.63-11.4) | 7.44 (5.76-11.82) | 7.69 (6.13-10.58) | *H*(4)=7.54, *P*=0.11 |
| N-acetylputrescine | 7.63 (5.92-23.52) | 5.92 (4.92-7.97) | 5.74 (4.46-7.34)^$^ | 5.95 (4.28-10.25) | 6.86 (5.58-9.63) | *H*(4)=12.98, *P*=0.011 |

^*^ *p* ≤ 0.05, ^**^*p* ≤0.01 and ^***^ *p* ≤ 0.001 *vs* 60-70 years old; ^$^ *p* ≤ 0.05 and ^$$^ *p* ≤ 0.01 *vs* 20-29 years old.
