# Supplementary material for: Age-associated polyamines in peripheral blood cells and plasma in 20 to 70 years of age subjects
Source: Amino Acids. 2023 Jun 13;55(6):789–98. doi: 10.1007/s00726-023-03269-2 (PMC10287822; doi:10.1007/s00726-023-03269-2)
Supplement: Supplementary file 2 — Supplementary file2 (DOCX 28 KB) [file 726_2023_3269_MOESM2_ESM.docx]

**Supplementary Table 2** Participants and polyamines content in samples of peripheral blood. Number (*n*) of participants, median of age in years and values of polyamines (nmol or pmol/mg of protein) (Percentile 25th-75th) by sex (Male and Female) in peripheral blood cells and plasma in each categorical group by decades.

| **Groups (years)** | **20-29** | **30-39** | **40-49** | **50-59** | **60-70** |
| --- | --- | --- | --- | --- | --- |
| ***n* (Gender: Male/Female)** | 41 (10/31) | 39 (15/24) | 40 (28/12) | 36 (22/14) | 37 (21/16) |
| **Age** (Years) Male  Age (Years) Female | 23 (21.5-24.25) 24 (21-27) | 35 (34-38) 36.5 (33.25-38) | 44.5 (42.25-47) 46 (45-46.75) | 55.5 (53.75-58)^††^ 51.5 (50.75-53.57) | 63 (62-65.5) 62.11 (60.25-67.86) |
| **Mononuclear Cells (nmol/mg of protein)** | |  |  |  |  |
| Putrescine Male  Putrescine Female | 1.09 (1.02-1.38)  0.95 (0.68-1.39) | 0.95 (0.75-1.48) 1.11 (0.77-1.74) | 0.99 (0.68-1.25) 1.03 (0.81-1.32) | 0.97 (0.65-1.17) 0.73 (0.51-0.99) | 0.76 (0.69-0.95) 0.77 (0.52-1.14) |
| Spermidine Male  Spermidine Female | 4.94 (4.41-6.18)  5.02 (3.57-6.61) | 4.3 (3.2-5.69) 4.29 (3.53-6.38) | 4.87 (3.57-5.98) 4.06 (3.57-5.72) | 3.79 (3.22-4.74) 3.98 (2.93-4.94) | 3.87 (3.43-5.18) 3.52 (2.72-4.76) |
| Spermine Male  Spermine Female | 10.28 (7.82-11.55)  8.39 (5.71-12.58) | 8.84 (6.26-12.2) 9.29 (7.33-12.13) | 9.35 (6.97-12.91) 7.61 (6.86-13.95) | 7.21 (5.25-8.86) 7.89 (5.97-9.29) | 7.28 (5.48-8.42) 6.94 (5.37-8.66) |
| N-acetylputrescine Male  N-acetylputrescine Female | 5.21 (1.87-25.18) 2.4 (1.33-4.23) | 1.85 (1.37-2.69) 2.19 (1.94-3.66) | 1.91 (1.59-2.31) 2.41 (1.46-3.68) | 1.85 (1.68-2.36) 1.86 (1.53-5.67) | 1.81 (1.15-3.05) 1.6 (1.08-2.71) |
| **Erythrocytes (pmol/mg of protein)** | |  |  |  |  |
| Putrescine Male  Putrescine Female | 8.43 (5.77-28.72) 12.58 (5.41-18.58) | 13.26 (10.34-30.61) 18.85 (10.72-34.99) | 22.24 (10.98-29.49) 16.18 (11.81-23.86) | 22.92 (15.24-31.22) 24.7 (11.15-32.02) | 4.8 (3.79-5.57) 5.66 (3.98-13.37) |
| Spermidine Male  Spermidine Female | 80.86 (32.99-124.12)^†††^ 155.3 (105-195.32) | 97.18 (77.08-114.22) 116.4 (86.24-144.45) | 109.93 (91.74-127.92) 125.98 (105-164.41) | 115.47 (96.4-151.99)^†^ 160.77 (119.24-230.95) | 126.31 (106.94-174.65) 134.03 (98.3-144.06) |
| Spermine Male  Spermine Female | 38.87 (29.24-63.19) 59.51 (41.55-82.04) | 57.91 (41.35-70.53) 62.7 (47.26-102.26) | 52.6 (38.5-71.41) 51.6 (38.39-82.88) | 48.95 (39.38-64.39) 73.31 (37.57-104.9) | 45.49 (32.07-68.4) 46.07 (35.14-106.41) |
| N-acetylputrescine Male  N-acetylputrescine Female | 33.32 (25.67-383.68) 54.78 (35.19-198.26) | 31.44 (28.19-39.97) 36.07 (28.94-43.03) | 32.74 (28.32-36.44)^†^ 42.35 (30.56-176.84) | 36.8 (26.44-48.49) 48.05 (31.28-289.24) | 45.58 (30.92-111.79) 39.13 (34.29-52.91) |
| **Plasma (pmol/mg of protein)** | |  |  |  |  |
| Putrescine Male  Putrescine Female | 21.88 (16.26-65.8) 17.75 (13.5-26.91) | 28.74 (20.08-37.51) 24.76 (16.74-29.43) | 24.75 (15.26-29.17)  16.16 (12.93-36.5) | 26.93 (15.74-34.45) 14.48 (13.07-23.08) | 8.41 (7.11-16.56) 8.3 (7-13.88) |
| Spermidine Male  Spermidine Female | 11.32 (4.53-14.61) 9.83 (8-13.3) | 6.92 (5.51-7.79) 6.23 (5.3-8.61) | 7.27 (6.19-8.98) 6.42 (5.5-8.07) | 7.72 (5.36-11.85) 6.51 (4.09-7.71) | 8.39 (4.98-16.18) 7.72 (4.31-11.94) |
| Spermine Male  Spermine Female | 2.69 (2.06-4.36) 4.79 (2.32-6.28) | 3.48 (2.94-5.15) 3.63 (2.83-4.94) | 3.54 (2.98-4.88) 4.95 (3.51-6.25) | 3.51 (3.02-5.1) 3.51 (2.14-5.1) | 2.15 (1.68-3.56) 2.9 (1.77-4.76) |
| N-acetylputrescine Male  N-acetylputrescine Female | 488.77 (317.92-829.81) 587.1 (363.3-853.48) | 630.69 (483.16-714.8) 576.67 (466.34-603.71) | 614.84 (526.83-753.62) 563.98 (458.66-781.16) | 617.54 (519.41-712.33) 573.68 (439.44-647.46) | 326.94 (275.62-502.3) 315.45 (269.68-476.41) |

^†^ *p* ≤ 0.05, ^††^ *p* ≤ 0.01 and ^†††^ *p* ≤ 0.001 comparing males *vs* females by means of Mann Whitney *U*-test.
