# Supplementary material for: Age-associated polyamines in peripheral blood cells and plasma in 20 to 70 years of age subjects
Source: Amino Acids. 2023 Jun 13;55(6):789–98. doi: 10.1007/s00726-023-03269-2 (PMC10287822; doi:10.1007/s00726-023-03269-2)
Supplement: Supplementary file 3 — Supplementary file3 (DOCX 43 KB) [file 726_2023_3269_MOESM3_ESM.docx]

**Supplementary Table 3** Unstandardized and standardized regression coefficients for the variables entered into the model. Polyamines content in blood cells and plasma that were significantly correlated and entered as predictors into a multiple regression using the standard method.

|  | **Unstandardized Coefficients** | | **Standardized Coefficients** |  | **Significance** |
| --- | --- | --- | --- | --- | --- |
| **Predictors** | **B** | **Std. Error** | **β** | ***t*** | ***p-*value** |
| **20-70 years** | | |  | |  |
| *Adjusted R^2^=0.133; ANOVA=F(3,168)=9.74, p < 0.001* | |  |  |  |  |
| **Constant** | 61.52 | 3.609 |  | 17.047 | <0.001 |
| **Spermidine in mononuclear cells (nmol/mg protein)** | -1.895 | 0.588 | -0.232 | -3.224 | 0.002 |
| **Putrescine in erythrocytes (pmol/mg protein)** | -0.161 | 0.08 | -0.148 | -2.015 | 0.046 |
| **Putrescine in plasma (pmol/mg protein)** | -0.28 | 0.08 | -0.256 | -3.519 | <0.001 |
|  | | |  | |  |
| **30-70 years** | | |  | |  |
| *Adjusted R^2^=0.352; ANOVA=F(4,125)=18.55 p < 0.001* | |  |  |  |  |
| **Constant** | 66.295 | 2.632 |  | 25.192 | <0,001 |
| **Putrescine in erythrocytes (pmol/mg protein)** | -0.271 | 0.071 | -0.348 | -3.814 | <0,001 |
| **Spermine in plasma (pmol/mg protein)** | -0.878 | 0.342 | -0.234 | -2.569 | 0.012 |
| **Spermidine in plasma (pmol/mg protein)** | 0.344 | 0.138 | 0.22 | 2.498 | 0.014 |
| **Spermidine in mononuclear cells (nmol/mg protein)** | -1.111 | 0.467 | -0.199 | -2.377 | 0.02 |
| **Putrescine in plasma (pmol/mg protein)** | -0.132 | 0.065 | -0.191 | -2.026 | 0.046 |
|  | | |  | |  |
| **40-70 years** | | |  | |  |
| *Adjusted R^2^=0.398; ANOVA=F(5,88)=13.28 p < 0.001* | | |  |  |  |
| **Constant** | 66.295 | 2.632 |  | 25.192 | <0,001 |
| **Putrescine in erythrocytes (pmol/mg protein)** | -0.271 | 0.071 | -0.348 | -3.814 | <0.001 |
| **Spermine in plasma (pmol/mg protein)** | -0.878 | 0.342 | -0.234 | -2.569 | 0.012 |
| **Spermidine in plasma (pmol/mg protein)** | 0.344 | 0.138 | 0.22 | 2.498 | 0.014 |
| **Spermidine in mononuclear cells (nmol/mg protein)** | -1.111 | 0.467 | -0.199 | -2.377 | 0.02 |
| **Putrescine in plasma (pmol/mg protein)** | -0.132 | 0.065 | -0.191 | -2.026 | 0.046 |
|  | |  |  |  |  |
| **50-70 years** | |  |  |  |  |
| *Adjusted R^2^=0.45; ANOVA=F(1,57)=48.41 p < 0.001* | | |  |  |  |
| **Constant** | 64.222 | 0.926 |  | 69.349 | <0.001 |
| **Putrescine in erythrocytes (pmol/mg protein)** | -0.354 | 0.051 | -0.678 | -6.958 | <0.001 |

*R^2^* = adjusted value of *r*; ANOVA: Analysis of Variance.
