# Supplementary material for: Age-associated polyamines in peripheral blood cells and plasma in 20 to 70 years of age subjects
Source: Amino Acids. 2023 Jun 13;55(6):789–98. doi: 10.1007/s00726-023-03269-2 (PMC10287822; doi:10.1007/s00726-023-03269-2)
Supplement: Supplementary file 4 — Supplementary file4 (DOCX 39 KB) [file 726_2023_3269_MOESM4_ESM.docx]

**Supplementary Table 4 Polyamines content in whole blood, blood cells and plasma of peripheral blood.** Median (Percentile 25th-75th) of the polyamines content in whole blood, mononuclear cells, erythrocytes, and plasma in 20-29 and 60-70 years of age subjects.

| **Groups (years)** | **20-29** | **60-70** | **Mann-Whitney *U*-test** |
| --- | --- | --- | --- |
| ***n*, Gender (M/F)** | 8 (3/5) | 12 (9/3) |  |
| **Age (Years)** | 24 (20.75-24.75) | 65.5 (62.25-68) |  |
| **Whole Blood (nmol/ml)** | | |  |
| Putrescine | 0.028 (0.022-0.031) | 0.022 (0.015-0.024) | *U=*27, *p =* 0.115 |
| Spermidine | 0.51 (0.46-0.54) | 0.41 (0.32-0.49) | *U=*24,  *p =* 0.069 |
| Spermine | 0.21 (0.2-0.22) | 0.25 (0.12-0.31) | *U=*51,  *p =* 0.850 |
| **Mononuclear cells (nmol/mg of protein)** | | | |
| Putrescine | 0.96 (0.42-1.42) | 0.74 (0.57-0.95) | *U=*42,  *p =* 0.678 |
| Spermidine | 4.84 (3.97-7.39) | 3.68 (2.81-3.88) | *U=*20,  *p =* 0.050 |
| Spermine | 6.54 (4.83-10.44) | 5.68 (4.93-7.28) | *U=*40,  *p =* 0.571 |
| **Erythrocytes (pmol/mg of protein)** | | | |
| Putrescine | 5.58 (4.1-6.7) | 4.22 (3.74-4.61) | *U=*22,  *p =* 0.047 |
| Spermidine | 160.57 (124.2-180.95) | 120.49 (106.56-137.7) | *U=*21,  *p =* 0.038 |
| Spermine | 38.47 (35-51.47) | 39.12 (29.27-50.69) | *U=*46,  *p =* 0.910 |
| **Plasma (pmol/mg of protein)** | | | |
| Putrescine | 9.47 (6.95-17.91) | 7.23 (6.17-7.84) | *U=*25,  *p =* 0.082 |
| Spermidine | 12.95 (12.16-15.25) | 7.62 (4.88-26.75) | *U=*38,  *p =* 0.657 |
| Spermine | 2.07 (1.94-2.34) | 1.94 (1.76-2.38) | *U=*38,  *p =* 0.472 |
